# Symptoms of Nomophobia, Psychological Aspects, Insomnia and Physical Activity: A Cross-Sectional Study of ESports Players in Saudi Arabia

**DOI:** 10.3390/healthcare10020257

**Published:** 2022-01-28

**Authors:** Mezna A. AlMarzooqi, Omar A. Alhaj, Maha M. Alrasheed, Mai Helmy, Khaled Trabelsi, Ahmed Ebrahim, Suhaib Hattab, Haitham A. Jahrami, Helmi Ben Saad

**Affiliations:** 1 Department of Community Health Sciences, College of Applied Medical Sciences, King Saud University, Riyadh 11451, Saudi Arabia; 2 Department of Nutrition, Faculty of Pharmacy and Medical Sciences, University of Petra, Amman 961343, Jordan; Omar.alhaj@uop.edu.jo; 3 Clinical Pharmacy Department, College of Pharmacy, King Saud University, Riyadh 11451, Saudi Arabia; mahalrasheed@KSU.EDU.SA; 4 Psychology Department, College of Education, Sultan Qaboos University, Muscat 123, Oman; m.helmy@squ.edu.om; 5 Psychology Department, Faculty of Arts, Menoufia University, Shebin El-Kom 32511, Egypt; 6 High Institute of Sport and Physical Education of Sfax, University of Sfax, Sfax 3000, Tunisia; trabelsikhaled@gmail.com; 7 Research Laboratory: Education, Motricity, Sport and Health, EM2S, LR19JS01, University of Sfax, Sfax 3000, Tunisia; 8 Ministry of Health, Manama 410, Bahrain; ahh50@hotmail.com (A.E.); hjahrami@health.gov.bh (H.A.J.); 9 Division of Physiology, Pharmacology & Toxicology, Department of Biomedical Sciences, Faculty of Medicine and Health Sciences, AnNajah National University, Nablus 4000, Palestine; suhaib.hattab@najah.edu; 10 College of Medicine and Medical Sciences, Arabian Gulf University, Manama 329, Bahrain; 11 Heart Failure (LR12SP09) Research Laboratory, Farhat HACHED Hospital, Sousse 4000, Tunisia; helmi.bensaad@rns.tn; 12 Laboratory of Physiology, Faculty of Medicine of Sousse, University of Sousse, Sousse 4000, Tunisia

**Keywords:** electronic sport, food addiction, nomophobia, psychological condition, physical activity

## Abstract

(1) Background: ESports is a new trend of sports, which has gained considerable popularity worldwide. There is a scarcity of evidence that focuses on the lifestyle of ESports players (eSP) particularly on symptoms of nomophobia, level of anxiety, sleep quality, food consumption and physical activity. (2) Objective: to determine the prevalence and relationship between symptoms of nomophobia, psychological aspects, insomnia and physical activity of eSP in Saudi Arabia. (3) Methods: A cross-sectional study was conducted between March and April 2021 using a convenient self-selection adult sample. A total of 893 (216 eSP vs. 677 non-eSP (NeSP)) participants aged over 18 years were included. All participants answered a seven-part validated questionnaire that included: (i) sociodemographic questions; (ii) a symptoms of nomophobia questionnaire; (iii) general anxiety disorder questions, (iv) an insomnia severity index, (v) an Internet addiction scale, (vi) the Yale food addiction scale 2.0 short form and (vii) an international physical activity questionnaire. (4) Results: Among the entire population, the prevalence of moderate to severe nomophobia, anxiety, insomnia, Internet addiction and low physical activity were 29.8%, 13.9%, 63.3%, 27% and 2.8%, respectively. The eSP and NeSP differed significantly in nomophobia scale, anxiety and insomnia values. Compared to NeSP, eSP had a higher level of severe nomophobia *p* = 0.003, a severe level of anxiety *p* = 0.025 and symptoms of insomnia *p* = 0.018. Except for food addiction and physical activity, a positive correlation was identified between symptoms of nomophobia, anxiety and insomnia among eSP. (5) Conclusion: This study reported high prevalence of nomophobia, anxiety and insomnia among eSP compared to NeSP.

## 1. Introduction

ESports is a new trend of sports, which has gained considerable popularity as evidenced by hundreds of major ESports events from 2017 to 2021 with an annual viewership of 380 million people [1]. The recognition of ESports continuous to soar on a global scale, in which players are predominantly younger individuals [2]. ESports is described as an organized competitive play of various digital games [3]. Electronic sports (also known as e-sports or eSports) are a form of video game competition [3]. ESports has several fields with different rules and mechanics that have to be mastered by the ESports players (eSP) or so-called E-athletes. These fields or genres include player vs. player, Real-Time Strategy, First Person Shooter and Multiplayer Online Battlefield Arena [4]. ESports are often organized multiplayer video game competitions, often between professional players [3,5]. In every field, eSP have to continuously practice to achieve and maintain their optimum ability, skills and performance [5]. Globally, there are thousands of professional eSP [5]. In comparison with a casual gamer who plays for fun, entertainment and recreation, an eSP is a professional player who defines gaming as their job and who plays for competition [6]. The average compensation of an eSP varies from a professional athlete. According to an ESport website, a top eSP earned about USD 2.5–3.5 million in 2017 [7]. In video game culture, organized competitions have long been a common occurrence, but participants were primarily amateurs and therefore the term eSP is not to be used exclusively for professional eSP [7].

Similarly, to athletes, eSP undertake extensive training to hone different skills, such as hand-eye coordination, the improvement of fast reaction times and the process of rapid decision making that are needed in ESports games [8]. eSP are engaged in number of hours of training to improve game tactics, communication and movement precision [8]. For example, an eSP in Germany plays an average of 25 h a week [9]. Considering the increasing popularity of ESports and the intensity of training for eSP, this may negatively impact their health and lead to potential adverse health effects [5,8]. eSP need several hours of sitting in the same position in front of a computer, requiring visual attention and enduring a high level of stress, which closely equates to a sedentary lifestyle [5]. ESports are dominated by personal computer (PC) games; however, there has been a rising popularity of ESports in mobile gaming in which video games are played online via smartphones [10,11]. There can be a prize pool of around USD 300 thousand–8 million in different mobile Esports tournaments [11]. Several major Esports tournaments have occurred in different countries, particularly in Asian regions. For example, *PlayerUnknown’s Battleground: Mobile*, *Mobile legends: Bang Bang*, *Arena of Valor* and *League of Legends: Wild Rift* are the most popular mobile games that have been watched in ESport tournaments [12].

The engagement of mobile gaming is growing rapidly as more people choose to play games on their mobile phones. Despite video games being dominated by PC games, some people do not have a PC or gaming console. For this reason, mobile gaming reaches to an audience market higher than PC games. Although there are many benefits of mobile phones to users, there are also several adverse effects of smartphone usage [13,14,15]. For example, excessive smartphone use could lead to different adverse effects on physical and mental health, such as sleep quality, stress, aggression and hostility [13,14,15]. Empirical studies reported excessive screen time and video gaming are associated with increase abdominal obesity [16,17]. Furthermore, higher risk of sedentary lifestyle was also associated with video gaming due to the increased amount of time spent on them and inadequate physical activity [16]. Meanwhile, a study conducted in Turkey among teenagers interested in an ESports career revealed that excessive play and Esports’ competitive nature have significant effects on their psychological well-being [18].

According to a study of female university students in Dammam, nearly two-thirds of the participants had either problematic Internet use (38%) or Internet addiction (30%) [19]. Recent evidence reported an association between sleep quality and Internet addiction [20,21,22]. The association of Internet addiction with sleep quality and depressive symptoms has been reported in the literature [23]. Another study has associated negative health effects with frequent Internet use such as depression, distress and additional undesirable consequences, such as conflict, dishonesty, low performance and social isolation [24]. Yet another survey conducted among 221 adolescents highlighted an association of Internet addiction with depression and poor sleep quality [21]. Similarly, a study conducted in Turkey reported the relationship between Internet addiction, psychopathology and dysfunctional beliefs [25]. In contrast, Internet addiction and school involvement had a weak negative association and no link was found between gaming addiction and school engagement in Turkey [26].

Nomophobia is a term used to define an individual as having fear or worry of not having their smartphones or unable to use them. Previous studies in the Middle East showed a prevalence of nomophobia of 75% among 18–35-year-old [27]. In Gulf Cooperation Countries, Saudi Arabia ranked first regarding the proportion of smartphone users [28,29]. Furthermore, research suggests that individuals with high levels of nomophobia have irregular eating habits, low consumption of fruits and vegetables and high consumption of processed and fast food [30,31,32,33]. With the growing popularity of ESports worldwide, and given that it has some similarities with traditional sport, it is high time for an in-depth exploration of the healthy lifestyle of eSP. Our lives are made simpler by mobile phones, but they also bind us. Nomophobia involves not only bodily consequences, but also psychological and intellectual consequences. Sleep deprivation, worry, stress and sadness, all of which have been linked to Internet misuse, have also been linked to mobile phone overuse [30,31,32,33].

At present, there is a scarcity of evidence that focuses on anxiety, sleep quality, nomophobia, food consumption and physical activity among eSP. In addition, ESports participation and its competitive nature may lead to physical and psychological problems. Furthermore, there are still gaps that need to be filled regarding the relations of these factors, such as obesity, low levels of physical activity, anxiety and depression. The association between nomophobia and sleep quality has been described and mentioned above; however, the relation between anxiety, nomophobia and food addiction is still unexamined. We hypothesize that there is significant association between symptoms of nomophobia, psychological aspects, insomnia and physical activity of eSP. Therefore, this study was designed to determine the prevalence and relationship between symptoms of nomophobia, psychological aspects, insomnia and physical activity of eSP in Saudi Arabia. In addition, this study aimed to compare the difference of symptoms of nomophobia, psychological aspects, insomnia and physical activity between eSP and non-eSP (NeSP).

## 2. Methods

### 2.1. Study Design

We designed a multinational, cross-sectional study to address the current study objectives. This design is used to describe the associations between symptoms of nomophobia, anxiety, insomnia, Internet addiction, food addiction and physical activity. The current study used a non-probabilistic and convenience sample strategy among adults over 18 in Saudi Arabia. This study followed strengthening the reporting of observational studies in epidemiology (STROBE) guidelines to improve research design and reporting [34].

### 2.2. Study Setting, Samples, and Data Collection

Between March and April 2021, a consecutive self-selection adult sample method was used to conduct recruitment. The data was collected mainly through an online self-administered questionnaire from Saudi Arabia. The samples were collected from the general community based on the following inclusion criteria: (1) participants were over the age of 18 and capable of writing and speaking Arabic, (2) participants owned at least one mobile device and (3) participants volunteered to participate in the study and consented to disclose their information. Each participant with a mental disorder or a chronic medical condition, who participated in any clinical research or lifestyle modification program or who followed a diet plan were not included in the study. Participants were recruited for the study using electronic contact, word of mouth, and advertisements on social networking apps used by all authors implicated in this study such as WhatsApp, Signal, Viber, Messenger, Line and other social media platforms such as Facebook, Instagram and Twitter. eSP were mainly recruited from special interests’ groups connected over social media platforms (including Facebook, Instagram, Twitter and TikTok), while NeSP were recruited from the general population via crowdsourcing participants using instant messaging applications (including WhatsApp, Messenger, Telegram, Line et al.). All participants were asked if they participated in an ESport competition, and if they had a salary for playing games. eSP and NeSP were encouraged to forward the survey link to their contacts to obtain the maximum number of respondents. In this research, we define eSP as a player who has participated in an ESport competition, has a work contract or who received a salary for playing games in an ESport team and having game skills and status. A NeSP is a person who plays for entertainment and recreation, without an ESport work contract while having a game skills and status.

### 2.3. Instrumentation

Our study has seven key parts: (1) sociodemographic questions; (2) symptoms of nomophobia (nomophobia questionnaire (NMP-Q)); (3) general anxiety disorder (GAD); (4) insomnia severity index (ISI); (5) Internet addiction scale (IAS); (6) Yale food addiction scale 2.0 short form (YFAS 2.0 SF); and (7) international physical activity questionnaire (IPAQ).

The sociodemographic section gathered data on age, sex, body mass index (BMI), corpulence status, smoking habits, hours of Internet mobile used, total metabolic rate. The individuals’ BMIs were estimated using the participants self-reported weight and height. The following two additional questions were asked: have you participated in an ESport competition? Do you have in income in playing electronic games? Two groups of participants were identified: eSP and NeSP.

The NMP-Q is a self-administered scale to measure nomophobia levels in adults [35]. NMP-Q has been translated and tested in several language including the Arabic language [35]. The NMP-Q Arabic version [32], which has an outstanding reliability of 0.9 (overall Cronbach’s alpha coefficient), contains 20 items divided into four factors/subscales that correspond to the dimensions of nomophobia: (1) inability to communicate (6 items); (2) loss of connectedness (5 items); (3) inability to access information (4 items); and (4) giving up convenience (5 items). Each item is scored on a 7-point Likert scale. The overall score ranges from 20 to 140 points, and is divided into four nomophobia categories (no, mild, moderate, severe).

The third part of the survey assesses the GAD via a seven-item scale (GAD-7). The GAD-7 was developed to examine the GAD symptom criteria adopted from the diagnostic and statistical manual of mental disorders [36]. Each item was assessed on a 4-point Likert scale ranging from 0 (never) to 3 (almost every day) during the previous two weeks. The overall score range on the GAD-7 scale is 0 to 21, with 5–9 indicating mild anxiety symptoms, 10–14 indicating moderate anxiety symptoms, and 15 indicating severe anxiety symptoms. The reliability of GAD-7 in screening anxiety is reported in the Arabic language as highly consistent and with Cronbach α = 0.89 [37].

The fourth section of the survey is about the ISI, which is used to diagnose insomnia cases in adults over the previous month [38]. This is a seven-question test with excellent internal consistency (Cronbach alpha of 0.90) and includes a 5-point Likert scale for each item (falling asleep, staying asleep, early awakening, satisfaction, interference, noticeable, worrisome). The overall score ranges from 0 to 28, with the following categories: scores ranging from 0 to 7 indicate the absence of clinically significant insomnia; scores ranging from 8 to 14 indicate subthreshold insomnia; scores ranging from 15 to 21 indicate moderately serious clinical insomnia, and scores ranging from 21 to 28 indicate severe clinical insomnia. The Arabic validated ISI version was employed [39], which has strong psychometric qualities.

The IAS is the fifth part of the survey. This scale was created in 2004 [40] and later developed in 2010 [41]. IAS consists of 35 items assessed on a 5-point scale from 0 to 4, with 0 equaling never and 4 equaling very often. IAS uses four symptom clusters to diagnose Internet addiction: (1) withdrawal, (2) control problems, (3) functional impairment, and (4) social isolation. The instrument exhibits convergent validity and strong internal reliability (α = 0.94). The IAS has a high reliability on the Saudi population with reported alpha values of (0.74) and (0.78) [42,43].

The sixth part of the survey is related to the YFAS 2.0 SF, which was created in 2009 primarily to assess symptoms of addictive-like eating behavior and the difficulty in regulating food intake of such food types as candy, starches, salty snacks, fatty foods and other beverages such as sugary drinks [44]. The self-report, validated, modified and updated short forms of the Arabic version were used in the current study [45]. The Arabic version has 13 items, each of which is evaluated on an 8-point Likert scale from 0 to 7 on a range from “never” to “daily” over the last 12 months. The Arabic version has strong psychometric qualities as well as excellent reliability of 0.93 (Cronbach’s alpha coefficient).

The last part was the IPAQ, which was used to collect data related to physical activity [46,47,48]. The IPAQ collects information on three specific types of activities such as walking or light-, moderate- and vigorous-intensity activity over the previous 7 days. The physical activity time per week was allocated to activity energy expenditure values in metabolic equivalents (METs)-min/week [49]. The activity intensity ranges per METs equivalent: light (3.3 METs = walking), moderate (4.0 METs = cycling a < 10 mph), and vigorous (8.0 METs = running a 12 mile). For example, a person who walks for 20 min 5 days a week had a score of, 20 × 5 × 3.0 METs = 300 MET-minutes/week. The IPAQ was tested for reliability and validity in different countries and found to have acceptable measurement [48]. Previous studies have shown that IPAQ questionnaire was a reliable and valid for assessing physical activity among the Saudi population [50,51].

### 2.4. Ethics Approval

This research is part of a large multi-country project involving 22 Arabic countries. The research was reviewed and approved by Research Ethics Committee in Menofia University, Egypt (ID 21924220210630093911).

The participants were informed that participation in the survey was voluntary, and that they could opt out at any time. Participants agreed that the information they provided would be used and archived anonymously.

### 2.5. Data Analysis

Data analysis was carried out using Statistical Package for Social Sciences (SPSS) version 23 (SPSS Inc., Chicago, IL, USA). Data were processed using descriptive statistics and presented using frequencies, percentages for categorical variables and mean ± standard deviation for continuous variables. Pearson Chi-square test was used to compare two groups (eSP vs. NeSP). Pearson product-moment correlation-coefficient “Pearson r” was used to examine the correlations between the total score of nomophobia, Internet addiction, food addiction, anxiety, insomnia and physical activity. “Pearson r” was considered “high” when it was >0.70, “good” when it was between 0.50–0.70, “fair” if it was between 0.30–0.50 and “weak or no association” if it was <0.30 [52]. Multiple logistic regression analysis was used to examine the association between the study variables reporting 95% confidence interval, and *p*-value. All *p*-values were two-tailed, and a *p*-value of <0.05 was considered statistically significant.

## 3. Results

Of the 893 participants who participated in this study, 216 (24.2%) were eSP, and 677 (75.2%) were NeSP. Table 1 reports their general characteristics.

Table 2 presents the difference of demographic and clinical characteristics of eSP and NeSP. The two groups included similar percentages of participants having mild/moderate IAS, and vigorous/moderate/low physical activity. Compared to the NeSP group, the eSP group included higher percentages of participants with moderate or severe nomophobia, higher percentages of participants with mild or severe anxiety, and a higher percentage of participants with insomnia.

As shown in Table 3, there was a weak positive correlation between nomophobia, Internet addiction (r = 0.15), anxiety (r = 0.28) and insomnia (r = 0.25), (*p* = 0.001).

Table 4 reports the associations between nomophobia, Internet addiction, food addiction, anxiety and insomnia.

The analysis shows anxiety, insomnia and Internet addiction were positively associated with nomophobia. Conversely, both nomophobia and insomnia were positively associated with anxiety. In addition, nomophobia and anxiety were found to be significantly associated with measures of insomnia. As for Internet addiction as an outcome variable, the analysis found a significant positive association with nomophobia. No significant association was found between food addiction and other variables.

## 4. Discussion

This study assessed the associations between symptoms of nomophobia and psychological aspects such as anxiety, sleep quality (insomnia), Internet addiction, food addiction and physical activity of eSP in Saudi Arabia. The results revealed notable findings such as a significant difference in the proportion of eSP and NeSP in the determinants of nomophobia scale.

Given the pioneering character of our study, especially for the point related to nomophobia in eSP, results comparisons will be performed with studies evaluating the issue of nomophobia in students and/or young adults [27,28,53,54,55]. First, the higher frequency of severe nomophobia among eSP aligns with an earlier study revealing that 33.9% of medical students in Saudi Arabia presented with severe nomophobia [53]. Second, our findings are in parallel with previous studies in the Middle East countries reporting high severity of nomophobia among young adults and university students [27,28]. It seems that the severity of nomophobia and Internet addiction among eSP is common among the university students, particularly in Saudi Arabia. This can be related to the popularity of Internet-use in Saudi Arabia as confirmed by the high Internet addiction level of 50% among Al-Jouf university students, Saudi Arabia [54]. Furthermore, 12.4% were addicted to the Internet, and 57.9% had the potential to become addicted among medical students in Qassim University, Saudi Arabia [55].

The findings of this study highlight the psychological aspects of eSP in Saudi Arabia. Thus, the high frequency of severe anxiety among eSP indicates that Internet addiction leads to psychological issues such as severe anxiety. The competitive nature of ESports has been compared with traditional sports because it requires similar mental skills [56]. A previous study identified 11 mental skills (including utilizing pre-performance routines, adapting to competition and staying in the moment) of eSP to achieve optimal performance [57]. Furthermore, it has been demonstrated by the present findings that eSP suffer more frequently from insomnia compared to NeSP. Previous research has investigated the factors that cause sleep problems for eSP [58,59]. These studies reported that stress from the competition and prolonged exposure to blue light (high-energy light that comes from the screens) delay their sleep schedule [58,59]. The sleeping pattern of eSP may also be affected due to the intensive or congested training for a competition that compromises their ability to achieve optimal sleep. Even in traditional athletes, optimal sleep is important, as it is critical for cognitive functioning. As the ESports industry has grown and become more competitive, the findings suggests that there is a need for further research on the psychological determinants for eSP because it may lead to psychological problems.

This study reported a positive correlation between nomophobia, Internet addiction, anxiety and insomnia. Our findings are in line with the previous reports of an association between symptoms of nomophobia, insomnia and food addiction among young adults [60]. Similarly to the previous findings, no significant association was established between food addiction and symptoms of nomophobia and insomnia [60]. The findings also show that nomophobia was associated with several psychological aspects such as anxiety, insomnia and Internet addiction. This result was parallel to a previous study among medical students in Saudi Arabia in which high levels of stress and anxiety were observed among students with severe nomophobia [53]. Our findings also coincide with the results of the study conducted in Lebanon in which high levels of anxiety and insomnia were significantly associated with higher odds of having severe nomophobia [61]. The analysis for the path from nomophobia to insomnia, anxiety and Internet addiction, anxiety to insomnia and nomophobia, insomnia to nomophobia and anxiety were all highly significant. The analysis pointed out the direct effect of anxiety and insomnia on nomophobia among eSP. Meanwhile, the analysis shows no significant association between physical activity and symptoms of nomophobia, insomnia, anxiety, Internet addiction and food addiction. There is still conflicting evidence regarding the association of levels of physical activity with time spent playing video games. For example, a recent study exploring the relationship between sitting time, physical activity and BMI found that an individual’s sitting of more than 8 h per day were more likely to have lower activity participation and higher BMI [62]. In contrary, a review study found inconclusive evidence of the relationship between online multiplayer gameplay and negative consequences of physical health of players [63]. Given the findings of our study and the recent available evidence, more studies are needed to increase our understanding regarding the effects and associations of ESports video gaming and the physical health of individuals.

This study provides various key contributions that will fill the gap regarding the scarcity of literature on eSP in Saudi Arabia. The literature lacks studies discussing the psychological condition and nomophobic tendencies of eSP in Saudi Arabia. Since the Middle East region is one of the fastest-growing communities of eSP, and given the increasing popularity of Internet, it becomes important to examine the possibility that addictive Internet use might lead to nomophobic behaviors. The current study provides more understanding of the association of nomophobia and the psychological condition of eSP, which may help future researchers to examine nomophobic tendencies and recognize its possible connection.

Recent research showed that insomnia is associated with nomophobia, but not with age, gender, BMI or mobile phone screen size in young adults [64]. The research discussed variables that might be at play in the link between mobile device use and sleeplessness [64]. Blue light from smartphone displays, for example, interferes with the synthesis of melatonin, the master hormone that governs the circadian rhythm. According to a recent comprehensive review, 2 h of exposure to shortwave blue light with a wavelength of 400–450 nanometers is enough to severely suppress melatonin. After 15 min of no exposure to artificial light, melatonin levels begin to recover quickly [60].

The authors acknowledge some limitations of this study. First, the descriptive correlational design could not determine causality. Second, a confounding factor (i.e., gaming disorder (GD)) was not evaluated in this study. GD is a mental health issue, which intricates relationships with specific health-related factors and well-being [65]. A recent study including 474 participants aged 18–66 years reported that age, attention problems and physical health problems significantly predicted GD [64]. Within our eSP group, it is possible that some players would meet the criteria for GD, and therefore they are likely to score highly on the NMP-Q and probably IAS. Another limitation is that the data were derived from self-reports which may influence bias. Moreover, the limited nature of the questionnaires adopted in the current study is based on symptoms and not on diagnosis. For example, the food addiction scale could only provide symptoms of eSP, and clinical diagnosis can only be done through interviews of a healthcare professional. In addition, the level of diversification between the type of eSP and types of games was not recorded, and because console, PC and mobile player demographics differ this hindered the generalizability of the study. Lastly, the use of a convenience sample and non-random selection of the eSP and NeSP groups make the interpretation and generalizability of our findings throughout the country “debatable”. However, our findings may serve as additional literature that might be useful for future comparisons about the psychological condition of eSP, if it is possible. Our study may help in developing or enhancing intervention programs to support eSP in Saudi Arabia.

## 5. Conclusions

The present study reported high frequencies of nomophobia, anxiety and insomnia among eSP. A positive correlation was identified between symptoms of nomophobia, anxiety and insomnia among eSP. Food addiction was not present for both eSP and NeSP. Concerning Internet addiction, the analysis found a significant association only with nomophobia. Psychological interventions such as interactive or counseling group sessions might help lessen eSP distress during training and competition. In addition, a sleep intervention designed specifically among eSP (e.g., lessen the effect of blue light) might also help to prevent insomnia and achieve optimal sleep. Regarding the future research directions, assessment and evaluation of gaming addiction and GD within eSP is needed particularly in those times when they cannot access or play their usual mode of gaming. Further studies may also help to better understand the psychological condition of eSP and its relationship with the nature of games, duration of play and during competitions. Moreover, more studies are needed to determine the negative side of Internet addiction, since the Internet can take up a long time.

## Figures and Tables

**Table 1:** Demographic characteristic of the participants.

Variables		*n* = 893
Age (years)		M = 24.30, SD = 6.04
Age ranges (years)	18–25	501 (56.1%)
	26–30	207 (23.2%)
	≥31	185 (20.7%)
Male sex		657 (73.6%)
Height, cm		M = 171, SD = 8
Weight, kg		M = 72, SD =10
Body mass index (BMI, kg/m^2^)		M = 24.6, SD = 3.6
Corpulence status	Underweight (BMI < 18.5 kg/m^2^)	33 (3.7%)
	Normal (BMI: 18.5–24.9 kg/m^2^)	474 (53.1%)
	Overweight (BMI: 25.0–29.9 kg/m^2^)	322 (36.1%)
	Obese (BMI > 30.0 kg/m^2^)	64 (7.2%)
Hours of Internet Mobile used		M = 7.44, SD = 3.18
Smoking status (Yes)		148 (16.6%)
Total metabolic rate/min (MET/min)		M = 1752, SD = 666
MET categories (minutes/week)	Vigorous	M = 198, SD = 218
	Moderate	M = 989, SD = 575
	Low	M = 564, SD = 262

M: mean. MET: metabolic-equivalent-task. SD: Standard deviation.

**Table 2:** Difference of demographic and clinical characteristics between ESports (eSP, *n* = 216) and non-ESports (NeSP, *n* = 677) players.

Variable		Total (*n* = 893)	eSP	NeSP	*p*-Value
Age ranges (year)	18–25	501 (56.1)	127 (58.8)	374 (55.2)	0.512
	26–30	207 (23.2)	44 (20.4)	163 (24.1)	
	≥31	185 (20.7)	45 (20.8)	140 (20.7)	
Male sex		657 (73.6)	153 (70.8)	508 (74.4)	0.168
Smoking status (yes)		148 (16.6)	41 (19.0)	107 (15.8)	0.161
Corpulence status	Underweight	33 (3.7)	10 (4.6)	23 (3.4)	0.811
	Normal	474 (53.1)	113 (52.3)	361 (53.3)	
	Overweight	322 (36.1)	76 (35.2)	246 (36.3)	
	Obese	64 (7.2)	17 (7.9)	47 (6.9)	
Nomophobia	Absence	5 (0.6)	2 (0.9)	3 (0.4)	**0.003**
	Mild	157 (17.6)	20 (9.3)	137 (20.2)	
	Moderate	465 (52.1)	125 (57.9)	340 (50.2)	
	Severe	266 (29.8)	69 (31.9)	197 (29.1)	
Anxiety	Minimal	180 (20.2)	30 (13.9)	150 (22.2)	**0.025**
	Mild	350 (39.2)	97 (44.9)	253 (37.4)	
	Moderate	239 (26.8)	54 (25.0)	185 (27.3)	
	Severe	124 (13.9)	35 (16.2)	89 (13.1)	
Insomnia	No	328 (36.7)	66 (30.6)	262 (38.7)	**0.018**
	Yes	565 (63.3)	150 (69.4)	415 (61.3)	
Internet addiction scale	Mild	652 (73.0)	148 (68.5)	504 (74.4)	0.050
	Moderate	241 (27.0)	68 (31.5)	173 (25.6)	
Total MET	Low	25 (2.8)	7 (3.2)	18 (2.7)	0.898
	Moderate	633 (70.9)	153 (70.8)	480 (70.9)	
	High	235 (26.3)	56 (25.9)	179 (26.4)	

Data were *n* (%). Two-sided Chi-square test was used to compare the two groups (eSP vs. NeSP). Bold *p*-values (*p* < 0.05) considered significant.

**Table 3:** Pearson correlation “r” between total score of nomophobia, anxiety, insomnia, Internet addiction, food addiction and physical activity.

Variables	Means (SD)	r
Nomophobia	84.01 (26.45)	-
Anxiety	8.81 (5.01)	0.281 *
Insomnia	7.49 (4.40)	0.253 *
Internet addiction	46.96 (5.64)	0.158 *
Food addiction	1.95 (1.70)	−0.012
Physical activity	1751.67 (665.9)	−0.010

* Significant “r” at 0.01 level. All significant correlations were weak (“r” < 0.30).

**Table 4:** Association between nomophobia, anxiety and insomnia, Internet addiction, food addiction and physical activity.

Variable	β (95% Confidence Interval)	*p*-Value
Outcome Variable = Nomophobia		
Anxiety	1.03 (0.61–1.45)	**0.001**
Insomnia	0.70 (0.23–1.18)	**0.004**
Internet addiction	0.57 (0.28–0.87)	**0.001**
Food addiction	0.09 (−0.87–1.06)	0.842
Physical activity	0.01 (−0.01–0.05)	0.799
Outcome Variable = Anxiety		
Nomophobia	0.02 (0.01–0.03)	**0.001**
Internet addiction	0.01 (−0.30–0.06)	0.493
Food addiction	0.03 (−0.12–0.18)	0.687
Insomnia	0.65 (0.59–0.71)	**0.001**
Physical activity	0.03 (−0.01–0.07)	0.495
Outcome Variable = Insomnia		
Nomophobia	0.01 (0.04–0.02)	**0.004**
Internet addiction	0.03 (−0.01–0.07)	0.114
Food addiction	−0.11 (−0.25–0.16)	0.083
Anxiety	0.51 (0.46–0.56)	**0.001**
Physical activity	−0.03 (−0.1–0.16)	0.275
Outcome Variable = Internet addiction		
Nomophobia	0.02 (0.01–0.04)	**0.001**
Food addiction	−0.11 (−0.33–0.09)	0.287
Anxiety	0.03 (−0.06–0.12)	0.493
Insomnia	0.08 (−0.02–0.19)	0.114
Physical activity	−0.09 (−0.05–0.10)	0.738
Outcome Variable = Food Addiction		
Nomophobia	0.01 (−0.01–0.05)	0.835
Internet addiction	−0.01 (−0.31–0.09)	0.287
Anxiety	0.06 (−0.02–0.35)	0.687
Insomnia	−0.02 (−0.06–0.04)	0.083
Physical activity	−0.03 (−0.01–0.06)	0.291
Outcome Variable = Physical activity		
Nomophobia	0.22 (−1.97–1.52)	0.799
Internet addiction	3.90 (−7.31–15.11)	0.495
Anxiety	−4.39 (−12.28–3.49)	0.275
Insomnia	−2.16 (−14.85–10.53)	0.738
Food addiction	−13.86 (−29.62–11.90)	0.291

β: Standardized Coefficient. Bold *p*-value (<0.05) are considered significant.

## Data Availability

The data are available from the authors upon request.

## References

[1] Yin K., Zi Y., Zhuang W., Gao Y., Tong Y., Song L., Liu Y. (2020). Linking Esports to Health Risks and Benefits: Current Knowledge and Future Research Needs. J. Sport Health Sci..

[2] Chung T., Sum S., Chan M., Lai E., Cheng N. (2019). Will Esports Result in a Higher Prevalence of Problematic Gaming? A Review of the Global Situation. J. Behav. Addict..

[3] Witkowski E. (2012). On the Digital Playing Field: How We “Do Sport” with Networked Computer Games. Games Cult..

[4] Thiel A., John J.M. (2018). Is ESport a ‘Real’ Sport? Reflections on the Spread of Virtual Competitions. Eur. J. Sport Soc..

[5] Bányai F., Griffiths M.D., Király O., Demetrovics Z. (2019). The Psychology of Esports: A Systematic Literature Review. J. Gambl. Stud..

[6] Ma H., Wu Y., Wu X. (2013). Research on essential difference of E-sport and online game. Lecture Notes in Electrical Engineering.

[7] Insider E. MTG: Mobile Gaming is Unlocking a Whole New World of Esports. https://www.esportsinsider.com/2021/08/mtg-mobile-gaming-is-unlocking-a-whole-new-world-of-esports/.

[8] Nagorsky E., Wiemeyer J. (2020). The Structure of Performance and Training in Esports. PLoS ONE.

[9] Froboese I., Rudolf K., Wechsler K., Tholl C., Grieben C.E.S. (2019). ESportler im Fokus der Wissenschaft.

[10] Su Y.-S., Chiang W.-L., James Lee C.-T., Chang H.-C. (2016). The Effect of Flow Experience on Player Loyalty in Mobile Game Application. Comput. Hum. Behav..

[11] Tuting K. Top 10 Biggest Mobile Esports Prize Pool. https://www.oneesports.gg/gaming/biggest-mobile-esports-prize-pool/.

[12] Mobile Esports: Mobile Esports on the World Map: Asia in 2021. https://escharts.com/blog/mobile-esports-world-map-asia-2021.

[13] Clayton R.B., Leshner G., Almond A. (2015). The Extended ISelf: The Impact of IPhone Separation on Cognition, Emotion, and Physiology. J. Comput. Mediat. Commun..

[14] Elhai J.D., Dvorak R.D., Levine J.C., Hall B.J. (2017). Problematic Smartphone Use: A Conceptual Overview and Systematic Review of Relations with Anxiety and Depression Psychopathology. J. Affect. Disord..

[15] Hong F.-Y., Chiu S.-I., Huang D.-H. (2012). A Model of the Relationship between Psychological Characteristics, Mobile Phone Addiction and Use of Mobile Phones by Taiwanese University Female Students. Comput. Hum. Behav..

[16] Lam A.T.W., Perera T.P., Quirante K.B.A., Wilks A., Ionas A.J., Baxter G.D. (2020). E-Athletes’ Lifestyle Behaviors, Physical Activity Habits, and Overall Health and Wellbeing: A Systematic Review. Phys. Ther. Rev..

[17] Marker C., Gnambs T., Appel M. (2019). Exploring the Myth of the Chubby Gamer: A Meta-Analysis on Sedentary Video Gaming and Body Mass. Soc. Sci. Med..

[18] Kocadağ M. (2019). Investigating Psychological Well-being Levels of Teenagers Interested in Esport Career. Res. Educ. Psychol..

[19] Saquib J. (2020). Internet Addiction among Saudi Arabian Youth. Int. J. Health Sci..

[20] Nagori N., Vasava K., Vala A.U.V.U., Ratnani I.J. (2019). Association of Sleep Quality and Internet Addiction among the Medical Students. Int. J. Res. Med. Sci..

[21] Awasthi A.A., Taneja N., Maheshwari S., Gupta T. (2020). Prevalence of Internet Addiction, Poor Sleep Quality, and Depressive Symptoms among Medical Students: A Cross-Sectional Study. Osong Public Health Res. Perspect..

[22] Karimy M., Parvizi F., Rouhani M.R., Griffiths M.D., Armoon B., Fattah Moghaddam L. (2020). The Association between Internet Addiction, Sleep Quality, and Health-Related Quality of Life among Iranian Medical Students. J. Addict. Dis..

[23] Bhandari P.M., Neupane D., Rijal S., Thapa K., Mishra S.R., Poudyal A.K. (2017). Sleep Quality, Internet Addiction and Depressive Symptoms among Undergraduate Students in Nepal. BMC Psychiatry.

[24] Alqahtani A., Alqarni M., Alotaibi S., Fattah S., Alhalawany R. (2020). Relationship between Level of Internet Addiction, Loneliness and Life Satisfaction among College of Health and Rehabilitation Sciences Students’ at Princess NourahBint Abdulrahman University. Menoufia Nurs. J..

[25] Taymur I., Budak E., Demirci H., Akdağ H.A., Güngör B.B., Özdel K. (2016). A Study of the Relationship between Internet Addiction, Psychopathology and Dysfunctional Beliefs. Comput. Hum. Behav..

[26] Taş I. (2017). Relationship between Internet Addiction, Gaming Addiction and School Engagement among Adolescents. Univers. J. Educ. Res..

[27] Qutishat M., Rathinasamy Lazarus E., Razmy A.M., Packianathan S. (2020). University Students’ Nomophobia Prevalence, Sociodemographic Factors and Relationship with Academic Performance at a University in Oman. Int. J. Afr. Nurs. Sci..

[28] Ozdemir B., Cakir O., Hussain I. (2018). Prevalence of Nomophobia among University Students: A Comparative Study of Pakistani and Turkish Undergraduate Students. Eurasia J. Math. Sci. Technol. Educ..

[29] Naeem Z. (2014). Health Risks Associated with Mobile Phones Use. Int. J. Health Sci..

[30] Ibrahim N.K., Baharoon B.S., Banjar W.F., Jar A.A., Ashor R.M., Aman A.A., Al-Ahmadi J.R. (2018). Mobile Phone Addiction and Its Relationship to Sleep Quality and Academic Achievement of Medical Students at King Abdulaziz University, Jeddah, Saudi Arabia. J. Res. Health Sci..

[31] Al-Khlaiwi T., Meo S.A. (2004). Association of Mobile Phone Radiation with Fatigue, Headache, Dizziness, Tension and Sleep Disturbance in Saudi Population. Saudi Med. J..

[32] O’Donnell S., Epstein L.H. (2019). Smartphones Are More Reinforcing than Food for Students. Addict. Behav..

[33] Merlo L.J., Stone A.M., Bibbey A. (2013). Measuring Problematic Mobile Phone Use: Development and Preliminary Psychometric Properties of the PUMP Scale. J. Addict..

[34] von Elm E., Altman D.G., Egger M., Pocock S.J., Gøtzsche P.C., Vandenbroucke J.P. (2008). The Strengthening the Reporting of Observational Studies in Epidemiology (STROBE) Statement: Guidelines for Reporting Observational Studies. J. Clin. Epidemiol..

[35] Yildirim C., Correia A.-P. (2015). Exploring the Dimensions of Nomophobia: Development and Validation of a Self-Reported Questionnaire. Comput. Hum. Behav..

[36] Löwe B., Decker O., Müller S., Brähler E., Schellberg D., Herzog W., Herzberg P.Y. (2008). Validation and Standardization of the Generalized Anxiety Disorder Screener (GAD-7) in the General Population. Med. Care.

[37] Albagmi F.M., AlNujaidi H.Y., Al Shawan D.S. (2021). Anxiety Levels amid the COVID-19 Lockdown in Saudi Arabia. Int. J. Gen. Med..

[38] Morin C.M., Belleville G., Bélanger L., Ivers H. (2011). The Insomnia Severity Index: Psychometric Indicators to Detect Insomnia Cases and Evaluate Treatment Response. Sleep.

[39] Suleiman K.H., Yates B.C. (2011). Translating the Insomnia Severity Index into Arabic. J. Nurs. Scholarsh..

[40] Nichols L.A., Nicki R. (2004). Development of a Psychometrically Sound Internet Addiction Scale: A Preliminary Step. Psychol. Addict. Behav..

[41] Gunuc S., Kayri M. (2010). The Profile of Internet Dependency in Turkey and Development of Internet Addiction Scale: Study of Validity and Reliability. Hacet. Univ. J. Educ..

[42] Malik A.A., Bakarman M.A., Butt N.S. (2018). Construct Validity and Factor Structure of the Pittsburgh Sleep Quality Index (PSQI) among Physicians in Jeddah, Kingdom of Saudi Arabia. Pak. J. Stat. Oper. Res..

[43] Alghannami A., Alrashed A., Alshehri R., Alotaibi S., Alharbi M., Mukaddem A., Agha S. (2021). The Sleep Pattern of Medical Students: Examining the Impact of Excessive Internet Use. Int. J. Med. Dev. Ctries..

[44] Gearhardt A.N., Corbin W.R., Brownell K.D. (2009). Preliminary Validation of the Yale Food Addiction Scale. Appetite.

[45] Mobarak E., Eldeeb D., El-Weshahi H. (2019). Reliability of an Arabic Version of the Short Form Modified Yale Food Addiction Scale. J. High Inst. Public Health.

[46] Bowe A.K., Owens M., Codd M.B., Lawlor B.A., Glynn R.W. (2019). Physical Activity and Mental Health in an Irish Population. Ir. J. Med. Sci..

[47] Cleland C., Ferguson S., Ellis G., Hunter R.F. (2018). Validity of the International Physical Activity Questionnaire (IPAQ) for Assessing Moderate-to-Vigorous Physical Activity and Sedentary Behaviour of Older Adults in the United Kingdom. BMC Med. Res. Methodol..

[48] Craig C.L., Marshall A.L., Sjöström M., Bauman A.E., Booth M.L., Ainsworth B.E., Pratt M., Ekelund U., Yngve A., Sallis J.F. (2003). International Physical Activity Questionnaire: 12-Country Reliability and Validity. Med. Sci. Sports Exerc..

[49] Ainsworth B.E., Haskell W.L., Herrmann S.D., Meckes N., Bassett D.R., Tudor-Locke C., Greer J.L., Vezina J., Whitt-Glover M.C., Leon A.S. (2011). 2011 Compendium of Physical Activities: A Second Update of Codes and MET Values. Med. Sci. Sports Exerc..

[50] Mahfouz M.S., Ali S.A., Bahari A.Y., Ajeebi R.E., Sabei H.J., Somaily S.Y., Madkhali Y.A., Hrooby R.H., Shook R.N. (2020). Association between Sleep Quality and Physical Activity in Saudi Arabian University Students. Nat. Sci. Sleep.

[51] Alghamdi A.S., Alghamdi K.A., Jenkins R.O., Alghamdi M.N., Haris P.I. (2020). Impact of Ramadan on Physical Activity and Sleeping Patterns in Individuals with Type 2 Diabetes: The First Study Using Fitbit Device. Diabetes Ther..

[52] Witz K., Hinkle D.E., Wiersma W., Jurs S.G. (1990). Applied Statistics for the Behavioral Sciences. J. Educ. Stat..

[53] Bano N., Khan M.A., Asif U., de Beer J., Rawass H. (2021). Effects of Nomophobia on Anxiety, Stress and Depression among Saudi Medical Students in Jeddah, Saudi Arabia. J. Pak. Med. Assoc..

[54] Abdel-Salam D.M., Alrowaili H.I., Albedaiwi H.K., Alessa A.I., Alfayyadh H.A. (2019). Prevalence of Internet Addiction and Its Associated Factors among Female Students at Jouf University, Saudi Arabia. J. Egypt. Public Health Assoc..

[55] Taha M.H., Shehzad K., Alamro A.S., Wadi M. (2019). Internet Use and Addiction among Medical Students in Qassim University, Saudi Arabia. Sultan Qaboos Univ. Med. J..

[56] Poulus D., Coulter T.J., Trotter M.G., Polman R. (2020). Stress and Coping in Esports and the Influence of Mental Toughness. Front. Psychol..

[57] Himmelstein D., Liu Y., Shapiro J.L. (2017). An Exploration of Mental Skills among Competitive League of Legend Players. Int. J. Gaming Comput. Mediat. Simul..

[58] Bonnar D., Castine B., Kakoschke N., Sharp G. (2019). Sleep and Performance in Eathletes: For the Win!. Sleep Health.

[59] Bonnar D., Lee S., Gradisar M., Suh S. (2019). Risk Factors and Sleep Intervention Considerations in Esports: A Review and Practical Guide. Sleep Med. Res..

[60] Jahrami H., Abdelaziz A., Binsanad L., Alhaj O.A., Buheji M., Bragazzi N.L., Saif Z., BaHammam A.S., Vitiello M.V. (2021). The Association between Symptoms of Nomophobia, Insomnia and Food Addiction among Young Adults: Findings of an Exploratory Cross-Sectional Survey. Int. J. Environ. Res. Public Health.

[61] Farchakh Y., Hallit R., Akel M., Chalhoub C., Hachem M., Hallit S., Obeid S. (2021). Nomophobia in Lebanon: Scale Validation and Association with Psychological Aspects. PLoS ONE.

[62] Bullock V.E., Griffiths P., Sherar L.B., Clemes S.A. (2017). Sitting Time and Obesity in a Sample of Adults from Europe and the USA. Ann. Hum. Biol..

[63] Sublette V.A., Mullan B. (2012). Consequences of Play: A Systematic Review of the Effects of Online Gaming. Int. J. Ment. Health Addict..

[64] Jahrami H., Rashed M., AlRasheed M.M., Bragazzi N.L., Saif Z., Alhaj O., BaHammam A.S., Vitiello M.V. (2021). Nomophobia is Associated with Insomnia but Not with Age, Sex, BMI, or Mobile Phone Screen Size in Young Adults. Nat. Sci. Sleep.

[65] Moore S., Satel J., Pontes H.M. Investigating the Role of Health Factors and Psychological Well-Being in Gaming Disorder. Cyberpsychol. Behav. Soc. Netw..

